# 4-[(*E*)-(4-Methyl­phen­yl)imino­meth­yl]phenol

**DOI:** 10.1107/S1600536812007635

**Published:** 2012-02-29

**Authors:** L. Jothi, G. Vasuki, R. Ramesh Babu, K. Ramamurthi

**Affiliations:** a Department of Physics, NKR Government Arts College for Women, Namakkal 1, India; b Department of Physics, Kunthavai Naachiar Government Arts College (W) (Autonomous), Thanjavur 7, India; c Crystal Growth and Thin Film Laboratory, School of Physics, Bharathidasan University, Tiruchirappalli 24, India

## Abstract

In the title compound, C_14_H_13_NO, the two rings show significant deviation from coplanarity, with a dihedral angle between the two planes of 49.40 (5)°. The hy­droxy group is involved in an inter­molecular O—H⋯N hydrogen bond, forming an extended one-dimensional zigzag chain along (001).

## Related literature

For the applications of Schiff bases, see: Qian & Cui (2009 ▶). For related structures, see: Burgess *et al.* (1999 ▶); Kaitner & Pavlovic (1995 ▶); Li (2010 ▶); Li *et al.* (2008 ▶); Yeap *et al.* (1993 ▶); Zhang (2010 ▶). For bond geometry, see: Allen *et al.* (1987 ▶).
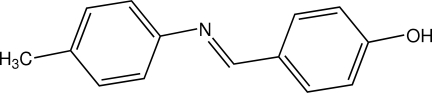


## Experimental

### 

#### Crystal data

C_14_H_13_NO
*M* *_r_* = 211.25
Orthorhombic, 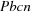

*a* = 21.618 (1) Å
*b* = 11.0561 (6) Å
*c* = 9.3318 (5) Å
*V* = 2230.4 (2) Å^3^
*Z* = 8
Mo *K*α radiation
μ = 0.08 mm^−1^
*T* = 296 K
0.30 × 0.20 × 0.20 mm

#### Data collection

Bruker Kappa APEXII CCD diffractometer
Absorption correction: multi-scan (*SADABS*; Bruker, 1999 ▶) *T*_min_ = 0.977, *T*_max_ = 0.984
11344 measured reflections
1961 independent reflections
1559 reflections with *I* > 2σ(*I*)
*R*_int_ = 0.028

#### Refinement

*R*[*F* ^2^ > 2σ(*F* ^2^)] = 0.036
*wR*(*F* ^2^) = 0.100
*S* = 1.08
1961 reflections
148 parameters
H-atom parameters constrained
Δρ_max_ = 0.19 e Å^−3^
Δρ_min_ = −0.14 e Å^−3^

### 

Data collection: *APEX2* (Bruker, 2004 ▶); cell refinement: *APEX2* and *SAINT* (Bruker, 2004 ▶); data reduction: *SAINT* and *XPREP* (Bruker, 2004 ▶); program(s) used to solve structure: *SHELXS97* (Sheldrick, 2008 ▶); program(s) used to refine structure: *SHELXL97* (Sheldrick, 2008 ▶); molecular graphics: *ORTEP-3* (Farrugia, 1997 ▶); software used to prepare material for publication: *PLATON* (Spek, 2009 ▶).

## Supplementary Material

Crystal structure: contains datablock(s) I, global. DOI: 10.1107/S1600536812007635/zs2179sup1.cif

Structure factors: contains datablock(s) I. DOI: 10.1107/S1600536812007635/zs2179Isup2.hkl

Supplementary material file. DOI: 10.1107/S1600536812007635/zs2179Isup3.cml

Additional supplementary materials:  crystallographic information; 3D view; checkCIF report

## Figures and Tables

**Table 1:** Hydrogen-bond geometry (Å, °)

*D*—H⋯*A*	*D*—H	H⋯*A*	*D*⋯*A*	*D*—H⋯*A*
O1—H1⋯N1^i^	0.88	1.87	2.7397 (17)	170

Symmetry code: (i) 
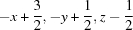
.

**Fig. 1.**
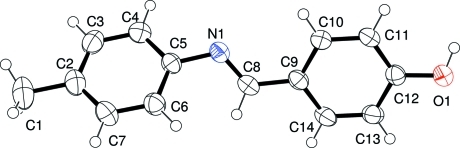
The molecular structure of the title compound showing atom numbering, with displacement ellipsoids drawn at the 50% probability level.

**Fig. 2.**
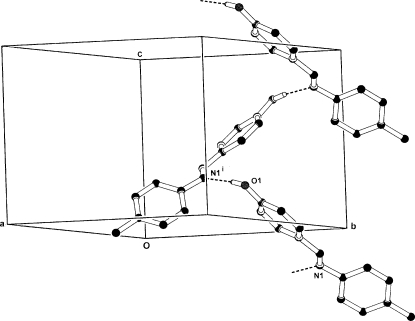
A perspective view of the one-dimensional chain structure in the title compound showing O—H···N interactions as dashed lines. For symmetry code (i), see Table 1.

## supplementary crystallographic information

### Comment

Schiff base compounds have attracted attention for the development of coordination chemistry related to catalysis and enzymatic reactions, magnetism and molecular architectures, e.g. (*E*)-2-methyl-*N*-[4-(methylsulfonyl)-benzylidene]aniline (Qian & Cui, 2009). As a part of our study on the coordination behaviour of ligands, an X-ray structural analysis of the title compound, C_14_H_13_NO (I) was carried out and the results are reported herein.

The molecule (I) (Fig. 1) may be described in terms of three planar subunits, namely two terminal benzene rings and their substituents bridged by a C═N imino moiety. The 4-hydroxybenzylidene system is nearly planar with r.m.s deviation of 0.0023 Å except for the hydroxy atom O1 which is 0.0183 Å out of the C9—C14 plane. The 4-methylbenzene system which is also essentially planar [r.m.s deviation, 0.0109 Å] except for the methyl atom C1 which is 0.0128Å out of the C2—C7 plane. The molecule has an *E*-configuration with respect to the C═N which is indicated by the torsion angle C9—C8—N1—C5 [-171.11 (13)°]. The twisting angles of the 4-hydroxybenzylidene and 4-methylbenzylidene groups with respect to the plane defined by the C5—N1—C8—C9 subunit [16.61 (15)° and 34.66 (10)°, respectively], are consistent with the general trend observed previously of aniline rings being more twisted than benzylidene rings, e.g. in 4-[(3-methoxyphenylimino)methyl]phenol [Yeap *et al.*, 1993] and *N*-*p*-tolylvanillaldimine [Kaitner & Pavlovic, 1995] and in four *N*-(2-hydroxybenzylidene)aniline derivatives [Burgess *et al.*, 1999]; 2-chloro-*N*-[4-(dimethylamino)benzylidene]aniline [Li *et al.*, 2008); 4-bromo-*N*-[4-(diethylamino)benzylidene]aniline [Li, 2010]; (4-chloro-*N*-[4-(diethylamino)benzylidene]aniline [Zhang, 2010]. The C9—C8 and N1—C5 bond distances [1.451 (2) and 1.4221 (19) Å] confirm π-electron delocalization between the benzene rings, and the molecule can be regarded as a partially delocalized π-electron system as observed in related structures (Yeap *et al.*, 1993; Kaitner & Pavlovic, 1995). In benzylideneaniline, where the phenyl ring has no substituents, the aromatic C—(C*sp*^2^), (C*sp*^2^) ═N and N—C_ar_ bond lengths of the azomethine portion are 1.496 (3), 1.237 (3) and 1.460 (3) Å, respectively (Kaitner & Pavlovic, 1995). If the terminal phenyl rings of benzylideneaniline have different substituents, the general pattern of two long and one short bond distance is not preserved. Contrary to this, the shortening of N—C_ar_ and aromatic C—(C*sp*^2^) [1.4221 (19) Å and 1.451 (2) Å. respectively] and the lengthening of N═(C*sp*^2^) [1.279 (2) Å] is observed in (I) and in similar structures (Yeap *et al.*, 1993; Kaitner & Pavlovic, 1995). In (I), the two longer bonds are also shortened, while the shorter bond has lengthened, compared to the parent compound. The C2—C1 bond distance of 1.504 (2) Å is in good agreement with the aromatic C—(C*sp*^3^) bond lengths. Using a 3σ criterion, the lengths of O1—C12 [1.3496 (18) Å] is the same and fall into the range for the O—C_ar_ bond type. Expansion of the exocyclic angle O1—C12—C11 [123.45 (14)°] may be due to the steric interaction atoms H11 and H1 [H1···H1 = 2.3029 (1) Å]. The N1—C8—C9 [124.80 (14)°] is greater than the normal value of 120°. This might be a consequence of repulsion between the lone pair of electrons on N1 and H10 attached to C10 [N1···H10 = 2.6892 (1) Å]. All other bond lengths are within the expected ranges (Allen *et al.*, 1987).

The crystal structure is stabilized by intermolecular hydroxy O—H···N hydrogen bonds (Table 1) linking the molecules into infinite one-dimensional chains extending along the *c* axis of the unit cell (Fig. 2).

### Experimental

The title compound (I) was prepared by mixing equimolar quantities (10 mmol) of 4-hydroxybenzaldehyde and 4-methylaniline in ethanol (40 ml). The reaction mixture was refluxed for about 6 h and the resulting solution was allowed to slowly evaporate at room temperature. After three days colourless single crystals of the title compound, suitable for X-ray structure analysis were obtained.

### Refinement

All of the H atoms were positioned geometrically and treated as riding on their parent atoms, with O—H = 0.88 Å, C—H = 0.93 Å (aromatic) or 0.96 Å (methyl), and refined using a riding model with *U*_iso_(H) = 1.2*U*_eq_(O or aromatic C) or 1.5*U*_eq_(methyl C).

### Crystal data

**Table:**

C_14_H_13_NO	*F*(000) = 896
*M**_r_* = 211.25	*D*_x_ = 1.258 Mg m^−^^3^
Orthorhombic, *P**b**c**n*	Mo *K*α radiation, λ = 0.71073 Å
Hall symbol: -P 2n 2ab	Cell parameters from 2333 reflections
*a* = 21.618 (1) Å	θ = 2.5–24.3°
*b* = 11.0561 (6) Å	µ = 0.08 mm^−^^1^
*c* = 9.3318 (5) Å	*T* = 296 K
*V* = 2230.4 (2) Å^3^	Needle, colourless
*Z* = 8	0.30 × 0.20 × 0.20 mm

### Data collection

**Table:**

Bruker Kappa APEXII CCD diffractometer	1961 independent reflections
Radiation source: fine-focus sealed tube	1559 reflections with *I* > 2σ(*I*)
Graphite monochromator	*R*_int_ = 0.028
ω and φ scans	θ_max_ = 25.0°, θ_min_ = 3.0°
Absorption correction: multi-scan (*SADABS*; Bruker, 1999)	*h* = −25→22
*T*_min_ = 0.977, *T*_max_ = 0.984	*k* = −13→13
11344 measured reflections	*l* = −9→11

### Refinement

**Table:**

Refinement on *F*^2^	Secondary atom site location: difference Fourier map
Least-squares matrix: full	Hydrogen site location: inferred from neighbouring sites
*R*[*F*^2^ > 2σ(*F*^2^)] = 0.036	H-atom parameters constrained
*wR*(*F*^2^) = 0.100	*w* = 1/[σ^2^(*F*_o_^2^) + (0.0452*P*)^2^ + 0.5906*P*] where *P* = (*F*_o_^2^ + 2*F*_c_^2^)/3
*S* = 1.08	(Δ/σ)_max_ < 0.001
1961 reflections	Δρ_max_ = 0.19 e Å^−^^3^
148 parameters	Δρ_min_ = −0.14 e Å^−^^3^
0 restraints	Extinction correction: *SHELXL97* (Sheldrick, 2008), Fc^*^=kFc[1+0.001xFc^2^λ^3^/sin(2θ)]^-1/4^
Primary atom site location: structure-invariant direct methods	Extinction coefficient: 0.0028 (10)

### Special details

**Table:**

Geometry. All e.s.d.'s (except the e.s.d. in the dihedral angle between two l.s. planes) are estimated using the full covariance matrix. The cell e.s.d.'s are taken into account individually in the estimation of e.s.d.'s in distances, angles and torsion angles; correlations between e.s.d.'s in cell parameters are only used when they are defined by crystal symmetry. An approximate (isotropic) treatment of cell e.s.d.'s is used for estimating e.s.d.'s involving l.s. planes.
Refinement. Refinement of *F*^2^ against ALL reflections. The weighted *R*-factor *wR* and goodness of fit *S* are based on *F*^2^, conventional *R*-factors *R* are based on *F*, with *F* set to zero for negative *F*^2^. The threshold expression of *F*^2^ > σ(*F*^2^) is used only for calculating *R*-factors(gt) *etc*. and is not relevant to the choice of reflections for refinement. *R*-factors based on *F*^2^ are statistically about twice as large as those based on *F*, and *R*-factors based on ALL data will be even larger.

### Fractional atomic coordinates and isotropic or equivalent isotropic displacement parameters (Å^2^)

**Table:**

	*x*	*y*	*z*	*U*_iso_*/*U*_eq_	
C1	0.98404 (9)	−0.3138 (2)	0.5166 (2)	0.0696 (6)	
H1A	1.0178	−0.2713	0.5605	0.104*	
H1B	0.9999	−0.3738	0.4519	0.104*	
H1C	0.9597	−0.3525	0.5894	0.104*	
C2	0.94434 (7)	−0.22573 (15)	0.43514 (19)	0.0463 (4)	
C3	0.93867 (8)	−0.10700 (16)	0.47974 (19)	0.0480 (4)	
H3	0.9599	−0.0814	0.5610	0.058*	
C4	0.90213 (7)	−0.02566 (14)	0.40601 (18)	0.0424 (4)	
H4	0.9001	0.0544	0.4363	0.051*	
C5	0.86863 (7)	−0.06207 (13)	0.28760 (16)	0.0344 (4)	
C6	0.87510 (8)	−0.18013 (14)	0.23975 (18)	0.0425 (4)	
H6	0.8543	−0.2055	0.1578	0.051*	
C7	0.91231 (8)	−0.25992 (15)	0.31352 (19)	0.0484 (5)	
H7	0.9160	−0.3390	0.2805	0.058*	
C8	0.78057 (7)	−0.01218 (13)	0.15734 (16)	0.0364 (4)	
H8	0.7683	−0.0915	0.1744	0.044*	
C9	0.74169 (7)	0.06129 (13)	0.06518 (16)	0.0343 (4)	
C10	0.76120 (7)	0.17174 (13)	0.00844 (16)	0.0348 (4)	
H10	0.7993	0.2033	0.0359	0.042*	
C11	0.72512 (7)	0.23468 (12)	−0.08724 (16)	0.0350 (4)	
H11	0.7389	0.3083	−0.1234	0.042*	
C12	0.66836 (7)	0.18910 (13)	−0.13011 (16)	0.0344 (4)	
C13	0.64808 (7)	0.07966 (13)	−0.07395 (18)	0.0407 (4)	
H13	0.6099	0.0486	−0.1011	0.049*	
C14	0.68428 (7)	0.01724 (13)	0.02146 (18)	0.0402 (4)	
H14	0.6702	−0.0561	0.0578	0.048*	
N1	0.83053 (6)	0.02412 (11)	0.21682 (13)	0.0350 (3)	
O1	0.63156 (5)	0.24423 (10)	−0.22698 (13)	0.0457 (3)	
H1	0.6475	0.3149	−0.2500	0.055*	

### Atomic displacement parameters (Å^2^)

**Table:**

	*U*^11^	*U*^22^	*U*^33^	*U*^12^	*U*^13^	*U*^23^
C1	0.0601 (12)	0.0750 (14)	0.0738 (14)	0.0191 (11)	−0.0089 (11)	0.0190 (11)
C2	0.0391 (9)	0.0503 (10)	0.0494 (10)	0.0064 (7)	0.0030 (8)	0.0115 (8)
C3	0.0432 (9)	0.0570 (11)	0.0438 (10)	−0.0041 (8)	−0.0069 (8)	0.0037 (8)
C4	0.0452 (9)	0.0386 (9)	0.0434 (9)	−0.0012 (7)	0.0005 (8)	−0.0019 (7)
C5	0.0370 (8)	0.0334 (8)	0.0330 (8)	0.0014 (6)	0.0036 (7)	0.0037 (6)
C6	0.0498 (9)	0.0379 (9)	0.0398 (9)	0.0047 (7)	−0.0027 (7)	−0.0021 (7)
C7	0.0541 (10)	0.0376 (9)	0.0535 (11)	0.0108 (8)	0.0018 (9)	0.0014 (8)
C8	0.0428 (8)	0.0282 (8)	0.0382 (9)	0.0015 (6)	0.0069 (7)	0.0025 (6)
C9	0.0389 (8)	0.0289 (7)	0.0350 (8)	0.0042 (6)	0.0044 (7)	−0.0007 (6)
C10	0.0367 (8)	0.0317 (8)	0.0360 (9)	−0.0007 (6)	0.0015 (7)	−0.0011 (6)
C11	0.0424 (8)	0.0260 (7)	0.0365 (9)	−0.0006 (6)	0.0034 (7)	0.0015 (6)
C12	0.0387 (8)	0.0301 (8)	0.0345 (9)	0.0066 (6)	0.0013 (7)	−0.0032 (6)
C13	0.0361 (8)	0.0322 (8)	0.0536 (11)	−0.0024 (7)	−0.0016 (8)	0.0000 (7)
C14	0.0424 (9)	0.0278 (8)	0.0503 (10)	−0.0011 (6)	0.0041 (8)	0.0045 (7)
N1	0.0403 (7)	0.0316 (7)	0.0332 (7)	0.0040 (5)	0.0017 (6)	0.0007 (5)
O1	0.0480 (7)	0.0368 (6)	0.0522 (7)	−0.0011 (5)	−0.0115 (6)	0.0080 (5)

### Geometric parameters (Å, º)

**Table:**

C1—C2	1.504 (2)	C8—N1	1.279 (2)
C1—H1A	0.9600	C8—C9	1.451 (2)
C1—H1B	0.9600	C8—H8	0.9300
C1—H1C	0.9600	C9—C14	1.394 (2)
C2—C7	1.382 (2)	C9—C10	1.396 (2)
C2—C3	1.383 (2)	C10—C11	1.375 (2)
C3—C4	1.381 (2)	C10—H10	0.9300
C3—H3	0.9300	C11—C12	1.386 (2)
C4—C5	1.381 (2)	C11—H11	0.9300
C4—H4	0.9300	C12—O1	1.3496 (18)
C5—C6	1.387 (2)	C12—C13	1.390 (2)
C5—N1	1.4221 (19)	C13—C14	1.372 (2)
C6—C7	1.378 (2)	C13—H13	0.9300
C6—H6	0.9300	C14—H14	0.9300
C7—H7	0.9300	O1—H1	0.8811
			
C2—C1—H1A	109.5	N1—C8—C9	124.80 (14)
C2—C1—H1B	109.5	N1—C8—H8	117.6
H1A—C1—H1B	109.5	C9—C8—H8	117.6
C2—C1—H1C	109.5	C14—C9—C10	117.61 (14)
H1A—C1—H1C	109.5	C14—C9—C8	119.57 (13)
H1B—C1—H1C	109.5	C10—C9—C8	122.63 (13)
C7—C2—C3	117.54 (15)	C11—C10—C9	121.17 (13)
C7—C2—C1	121.59 (17)	C11—C10—H10	119.4
C3—C2—C1	120.87 (17)	C9—C10—H10	119.4
C4—C3—C2	121.28 (16)	C10—C11—C12	120.39 (14)
C4—C3—H3	119.4	C10—C11—H11	119.8
C2—C3—H3	119.4	C12—C11—H11	119.8
C3—C4—C5	120.58 (15)	O1—C12—C11	123.45 (14)
C3—C4—H4	119.7	O1—C12—C13	117.38 (13)
C5—C4—H4	119.7	C11—C12—C13	119.16 (14)
C4—C5—C6	118.63 (14)	C14—C13—C12	120.19 (14)
C4—C5—N1	118.68 (13)	C14—C13—H13	119.9
C6—C5—N1	122.66 (14)	C12—C13—H13	119.9
C7—C6—C5	120.04 (16)	C13—C14—C9	121.47 (14)
C7—C6—H6	120.0	C13—C14—H14	119.3
C5—C6—H6	120.0	C9—C14—H14	119.3
C6—C7—C2	121.84 (16)	C8—N1—C5	118.71 (13)
C6—C7—H7	119.1	C12—O1—H1	109.5
C2—C7—H7	119.1		
			
C7—C2—C3—C4	0.3 (3)	C8—C9—C10—C11	174.95 (14)
C1—C2—C3—C4	−179.72 (16)	C9—C10—C11—C12	−0.3 (2)
C2—C3—C4—C5	2.0 (2)	C10—C11—C12—O1	−177.82 (13)
C3—C4—C5—C6	−3.5 (2)	C10—C11—C12—C13	0.7 (2)
C3—C4—C5—N1	178.62 (14)	O1—C12—C13—C14	177.90 (14)
C4—C5—C6—C7	2.7 (2)	C11—C12—C13—C14	−0.7 (2)
N1—C5—C6—C7	−179.48 (14)	C12—C13—C14—C9	0.3 (2)
C5—C6—C7—C2	−0.4 (3)	C10—C9—C14—C13	0.1 (2)
C3—C2—C7—C6	−1.1 (3)	C8—C9—C14—C13	−175.12 (14)
C1—C2—C7—C6	178.95 (17)	C9—C8—N1—C5	−171.11 (13)
N1—C8—C9—C14	−171.69 (15)	C4—C5—N1—C8	−147.79 (14)
N1—C8—C9—C10	13.4 (2)	C6—C5—N1—C8	34.4 (2)
C14—C9—C10—C11	−0.1 (2)		

### Hydrogen-bond geometry (Å, º)

**Table:**

*D*—H···*A*	*D*—H	H···*A*	*D*···*A*	*D*—H···*A*
O1—H1···N1^i^	0.88	1.87	2.7397 (17)	170

Symmetry code: (i) −*x*+3/2, −*y*+1/2, *z*−1/2.
